# *Berberis libanotica* Ehrenb Extract Shows Anti-Neoplastic Effects on Prostate Cancer Stem/Progenitor Cells

**DOI:** 10.1371/journal.pone.0112453

**Published:** 2014-11-07

**Authors:** Rabih El-Merahbi, Yen-Nien Liu, Assaad Eid, Georges Daoud, Leina Hosry, Alissar Monzer, Tarek H. Mouhieddine, Aline Hamade, Fadia Najjar, Wassim Abou-Kheir

**Affiliations:** 1 Department of Anatomy, Cell Biology and Physiological Sciences, Faculty of Medicine, American University of Beirut, Beirut, Lebanon; 2 Graduate Institute of Cancer Biology and Drug Discovery, College of Medical Science and Technology, Taipei Medical University, Taipei, Taiwan; 3 Faculty of Pharmacy, Lebanese University, Hadath, Lebanon; 4 Department of Biology, Faculty of Sciences II, Lebanese University, Fanar, Lebanon; 5 Department of Chemistry and Biochemistry, Faculty of Sciences II, Lebanese University, Fanar, Lebanon; Centro Cardiologico Monzino, Italy

## Abstract

Cancer stem cells (CSCs), including those of advanced prostate cancer, are a suggested reason for tumor resistance toward conventional tumor therapy. Therefore, new therapeutic agents are urgently needed for targeting CSCs. Despite the minimal understanding of their modes of action, natural products and herbal therapies have been commonly used in the prevention and treatment of many cancers. *Berberis libanotica* Ehrenb (BLE) is a plant rich in alkaloids which may possess anti-cancer activity and a high potential for eliminating CSCs. We tested the effect of BLE on prostate cancer cells and our data indicated that this extract induced significant reduction in cell viability and inhibited the proliferation of human prostate cancer cell lines (DU145, PC3 and 22Rv1) in a dose- and time-dependent manner. BLE extract induced a perturbation of the cell cycle, leading to a G0-G1 arrest. Furthermore, we noted 50% cell death, characterized by the production of high levels of reactive oxidative species (ROS). Inhibition of cellular migration and invasion was also achieved upon treatment with BLE extract, suggesting a role in inhibiting metastasis. Interestingly, BLE extract had a major effect on CSCs. Cells were grown in a 3D sphere-formation assay to enrich for a population of cancer stem/progenitor cells. Our results showed a significant reduction in sphere formation ability. Three rounds of treatment with BLE extract were sufficient to eradicate the self-renewal ability of highly resistant CSCs. In conclusion, our results suggest a high therapeutic potential of BLE extract in targeting prostate cancer and its CSCs.

## Introduction

Prostate cancer (PC) is the most commonly diagnosed non-cutaneous malignancy and the third most common cause of cancer mortality in the Western male population [1], [2]. Primary PC is androgen-dependent in nature and is usually treated with androgen deprivation therapy (ADT). Most frequently, however, hormonal therapy leads to recurrence in a few years and PC eventually progresses to an androgen-independent state or, a so-called castrate resistant PC (CRPC). CRPC is an aggressively metastatic and lethal form of PC and currently, there is no known effective treatment for it.

Prostate cancer stem cells (CSCs) share properties with normal stem cells as they tend to express high levels of: aldehyde dehydrogenase (ALDH) - a detoxifying enzyme - [3], multidrug resistance (MDR) efflux pumps and ABC transporters [4]–[6]. These defensive strategies render conventional therapy ineffective, due to the presence of fast proliferative cells in the tumor bulk and a great potential for sparing the putative cancer stem/progenitor cells [7]. In addition, it has been indicated that prostate CSCs do not express androgen receptors (AR) [8], [9] and may not respond to ADT as mature tumor cells do. Following ADT, cancer stem cells may frequently manage to repopulate the tumor mass with androgen-independent PC, which is an aggressively metastatic and lethal form of PC.

A wide range of strategies have been employed for the discovery of novel drugs that might carry beneficial effects for cancer patients. A targeted therapy is urgently needed to eradicate not only the cancer bulk, but also the CSC pool found within the tumor. Previous work in our laboratory has demonstrated the ability to enrich a population of PC stem/progenitor cells by growing them in 3D spheres-forming culture conditions, namely called prostaspheres [10], [11]. Several studies have recently shown that a number of bioactive food compounds may have an anti-CSC effect. For example, it has been recently reported that palm oil-extracted gamma-tocotrienol [12], polysaccharide-P (PSP), an active component extracted from the mushroom Turkey tail [13], and Genistein, a major isoflavone constituent of soybeans and soy products [14], exhibit potent inhibitory activity on protosphere formation ability and tumorigenicity of PC cells. These substances have been shown to target prostate CSCs *in vitro*, and suppress tumor formation *in vivo*. These findings highlight the potential use of herbal bioactive compounds for the production of therapeutic agents targeting CSC, either for the prevention or the treatment of PC.

*Berberis libanotica*, specifically its roots, has been used in traditional herbal Lebanese remedies for rheumatic and neuralgic diseases [15], [16]. *B. vulgaris* and *B. stata* are the two most studied species of *Berberis* [17]–[20]. Alkaloids constitute the major class of compounds reported to exist in *Berberis* species and represent a very wide range of secondary metabolites with important biological activities [21], [22]. Various herbal alkaloids exhibit *in vitro and in vivo* anti-proliferative and anti-metastatic effects on various types of cancers. Alkaloids, such as camptothecin [23] and vinblastine [24], have already been successfully developed into anti-cancer drugs. Berberine, a major alkaloid characterizing *Berberis* species, has been intensively investigated for its pharmacological properties. It was shown to inhibit the migration of melanoma cancer cells [25], and the growth of human tongue squamous carcinoma tumors in a murine xenograft model [26], enhance tumor necrosis factor-related apoptosis-inducing ligand in breast cancer [27], and exert a cytotoxic effect against many cell lines [25], [28], [29]. To date, the biological and phytochemical properties of *B. libanotica* extracts have only been investigated in two publications reporting the inhibition of adult T-cell leukaemia viability via ethanol fraction [30], and the inhibition of key enzymes linked to Alzheimer's disease [15]. However, little is known about the effect of BLE extract on PC and the proliferation and viability of CSCs. Therefore, the aim of this study is to investigate the therapeutic efficacy of *B. libanotica* root extract (via ammonia-dichloromethane extraction) in inhibiting the proliferation and reducing the viability of human PC cell lines grown in a conventional 2D monolayer model. Furthermore, this study will focus on an advanced assay (sphere formation assay) to investigate the effect of BLE extract in targeting an enriched population of prostate CSCs.

## Materials and Methods

### Cell Culture

Human prostate cancer cell lines, PC3, DU145, and 22Rv1 were obtained from the American Type Culture Collection (Manassas, VA), and were sent to us as a gift by Dr. Kathleen Kelly at the National Institutes of Health (Bethesda, MD). Cells were cultured in RPMI-1640 AQ medium (Sigma, USA) supplemented with 10% FBS (Sigma, USA), 1% penicillin-streptomycin (Sigma, USA) and 1% non-essential amino acids (Sigma, USA) and incubated at 37°C in a humidified incubator (95% air, 5% CO_2_). Cells were harvested by trypsin-EDTA at 37°C (Sigma, USA). Cells were pelleted, re-suspended and transferred into new-75 cm^2^ tissue culture flasks for maintenance, or seeded for experiments at a concentration of 1×10^4^ cells/cm^2^ for PC3 and Du145 and 1.5×10^4^ cells/cm^2^ for the 22Rv1 cell line.

### BLE Extraction

The roots of *Berberis libanotica* Ehrenb were collected in September 2011 from the Cedars area in North Lebanon (altitude 1400–1700 m; approximate GPS location: N 34°14'41"; E 36°2'15"). No specific permissions were required for these locations/activities as they did not involve endangered or protected species. The plant was identified by Pr. G. Tohmé (CNRS, Lebanon). The voucher specimen (code number BLCS12) was deposited in the herbarium of the Faculty of Sciences II, Lebanese University, Beirut, Lebanon.

The plant roots were shade-dried and powdered using an electric blender. The ammonia-dichloromethane extract was prepared as follows: 10 g of plant powder were moistened for 2 h with NH_4_OH solution followed by dichloromethane addition (100 ml). The mixture was macerated for 24 h under magnetic stirring, followed by filtration. The organic phase was concentrated at 40°C under reduced pressure and cryo-dried. The obtained extracts were kept in a cool place in dark containers. The rotary evaporator used was IKA RV 10 BASIC and the lyophiliser was LYOVAC GT4.

### MTT/Cytotoxicity Assay

Anti-proliferative and cytotoxic effects of BLE were measured *in vitro* via MTT ([3-(4, 5-dimethylthiazol-2-yl)-2, 5-diphenyltetrazolium bromide]) assays. DU145 and PC3 cells were seeded in 100 µl complete medium in 96-well culture plates at a density of 7500 cells/well and 22Rv1 cells at a density of 13250 cells per well. Cells were incubated overnight in the incubator and then treated in triplicates with various extract concentrations diluted in 100 µl complete media for 24, 48 and 72 h. In case of the ROS scavenger N-acetyl-L-cysteine (NAC; Sigma), DU145 cells were pretreated with 5 mM NAC for 30 minutes and cells were then treated with 30 µg/ml of BLE extract for 24 and 48 h as above. For each time point, 20 µl of 5 mg/ml - in 1 x phosphate buffered saline (PBS) -MTT reagent was added to each well and incubated at 37°C for 4 h. Finally, 100 µl of solubilization solution was added into each well to dissolve the formazan crystals. The reduced MTT optical density (OD) was measured at a wavelength of 595 nm using an ELISA reader (Multiskan Ex). The percentage of cell viability was presented as an OD ratio between the treated and untreated cells at the indicated concentrations.

### Trypan Blue Exclusion Method

Supernatants containing dead cells were collected and attached live cells were harvested by trypsin EDTA and added to the supernatant. The cell pellet was re-suspended in 100 µl media and 50 µl of cell suspension was mixed with 50 µl of trypan blue and then live/dead cells were counted using a hemocytometer.

### Cell Cycle Analysis by Flow Cytometry

DU145 and PC3 cells were seeded in duplicates into 6-well plates at a density of 1×10^5^ cells/well and were incubated for 24 h prior to drug treatment for 24, 48 or 72 h. Cells were then harvested, washed twice with PBS, centrifuged at 1500 rpm for 5 min at 4°C, re-suspended in 1 mL of cold PBS, fixed in 4 mL of cold absolute ethanol and then stored at −20C° until staining and analysis. Fixed cells were then treated for 1 h with 200 µg/ml DNase-free RNase A, stained with 1 mg/ml propidium iodide (PI) (Sigma, USA) and incubated for 10 min in the dark in a flow tube (BD Flacon). Fluorescence of PI, a measure of DNA content in a cell population, was done using flow cytometry (FACScan, Becton Dickinson). A total of 10,000 gated events were acquired in order to assess the proportions of cells in different stages of the cell cycle. Analysis of cell cycle distribution was performed using FlowJo Software.

### Lactate Dehydrogenase (LDH) Assay

The cytotoxicity of BLE extract on the DU145 cell line was determined by measuring the activity of lactate dehydrogenase (LDH) released from the cytosol of damaged cells using a Cytotoxicity Detection Kit PLUS (LDH) (Roche Diagnostics GmbH). The assay was conducted following the manufacturer's instructions. Briefly, medium supernatants of control and treated cells were collected and equal volume of reaction mixture was added. The reaction mixtures were incubated for 20 min at room temperature in the dark. Following incubation, the absorbance was determined at 490 nm using an ELISA plate reader. The percentage of cytotoxicity was calculated as: (exp.value - spontaneous control release) ÷ (max.control release - spontaneous control release) ×100.

### ROS detection by Nitroblue Tetrazolium Assay

DU145 cells were cultured in triplicate in a 96-well plate at a density of 1×10^4^ cells/cm^2^ and were then incubated for 24 h prior to BLE extracts treatment for 24, 48 or 72 h. In each condition, ROS production was evaluated by a nitroblue tetrazolium assay (NBT; Sigma). Culture media were aspirated and cells were incubated in PBS containing 1 mg/ml NBT at 37°C in the dark for 60 minutes. NBT was reduced by ROS to a dark-blue insoluble form of NBT called formazan that could be visualized. To quantify the formazan product, the intracellular formazan was solubilized in KOH 2 M and DMSO 5 M solution, and absorbance was determined at 630 nm using an ELISA plate reader.

### Apoptosis Assay

Apoptosis was assayed using the cellular DNA fragmentation ELISA (Roche Diagnostics GmbH, Mannheim, Germany) for the detection of BrdU-labeled DNA fragments in culture supernatants and cell lysates. The assay was used according to the manufacturer's protocol and as described previously [31]. Cells were incubated with 30 µg/ml of BLE extract for 48 h in the presence or absence of 5 mM N-acetyl-L-cysteine that was added 30 minutes before BLE treatment. Apoptosis in the control untreated cells was adjusted to 100% and the results were plotted as percent of control.

### Wound healing assay

For wound healing, or scratch assay, cells were seeded in a six-well plate with the same numbers, and incubated until they reached 80–90% confluence. Cells were then treated with 10 µg/ml mitomycin C (Sigma) for 2 h in order to block cellular proliferation. A sterile 200 µl tip was used to scratch wounds of the same width on each monolayer, the plates were then washed twice with PBS to remove the detached cells, and the remaining cells were cultured in complete media with or without treatment. Photos were subsequently taken at 0, 12, 30, and 48 h. The distance traveled by the cells into the wounded area enumerated the closure of the wounds. The experiment was repeated three times with duplicate measurements in each experiment.

### Sphere Formation Assay

In this study, all three cell lines, PC3, DU145 and 22Rv-1, were able to form spheroids in non-adherent culture, suggesting the presence of cancer stem-like cells within these cell lines. After counting the single cell suspension of prostate cancer cell lines, a concentration of 1,000 cells/well was suspended in cold Matrigel™/serum-free RPMI-1640 (1∶1) in a total volume of 50 µl. Cells were seeded uniformly in a circular manner around the bottom rim of a well in a 24-well plate and allowed to solidify in the incubator at 37°C for 45 minutes, before 0.5 ml of complete RPMI-1640 −2% FBS media (with or without treatment) was added gently in the middle of each well. Spheres were replenished with warm media as in the original seeding (with or without treatment) every two to three days. Spheres were counted after 13 days. To propagate spheres, the medium was aspirated and Matrigel™ was digested with 0.5 ml Dispase solution (Invitrogen, Carlsbad, CA, 1 mg/ml, dissolved in RPMI-1640 incomplete medium) for 60 minutes at 37°C. Spheres were collected, incubated in 1 ml warm Trypsin- EDTA at 37°C for 5 minutes, and then passed through a 27-gauge syringe 5 times. Cells were counted by a hemocytometer and re-seeded.

### Transwell Invasion Assay

For the invasion assay, 2.5×10^5^ cells were seeded in a serum-free medium with or without treatment in the top chamber onto the Matrigel™-coated membrane (24-well insert; pore size, 8 µm; Falcon), and a medium supplemented with serum was used as a chemo-attractant in the lower chamber. Each well was freshly coated with 100 µl of Matrigel™ (BD Bioscience) at a dilution of 1∶10 in cold PBS and was then air-dried overnight before starting the invasion assay. Cells were allowed to migrate through the membrane coated with Matrigel™ at 37°C in a 5% CO2 incubator for 24 and 48 h. Non-migratory cells in the upper chamber were then gently scraped off with a cotton-tip applicator. Invading cells on the lower surface of the membrane were fixed and stained with Hematoxylin and Eosin. After staining, the total number of invading cells was counted under the light microscope (×10 objective) from 6 consecutive fields for each well.

### RNA extraction and quantitative real time PCR (qRT-PCR)

Total RNA was extracted from cells, treated or non-treated with 30 µg/ml of BLE extract for 48 h, using the RNeasy Micro Kit (Qiagen) according to the manufacturer's instructions. cDNA was generated from total RNA using the Super Script III First Strand Synthesis System for RT-PCR (Invitrogen), and PCR was performed using Platinum Taq Polymerase (Invitrogen). For quantitative RT-PCR, the amplification step was done using the SYBR green PCR master mix (Applied Biosystems, Bedford, MA). All reactions were run in duplicate using the specific primers listed below and all values were normalized to the house keeping gene GAPDH. The primers used were: GAPDH-F: 5′ ACCTGGCTAGCGAAAAGCAA3′; GAPDH-R: 5′CCACTTTGTCAAGCTCATTTCCT3′; Sox2-F: 5′AACCCCAAGATGCACAACTC3′; Sox2-R: 5′GCTTAGCCTCGTCGATGAAC3′; Oct4-F: 5′AGAACATGTGTAAGCTGCGG3′; Oct4-R: 5′GTTGCCTCTCACTCGGTTC3′; Nanog-F: 5′ACCTCAGCTACAAACAGGTGAA3′; Nanog-R: 5′AAAGGCTGGGGTAGGTAGGT3′; CD44-F: 5′TTTGCATTGCAGTCAACAGTC3′; CD44-R: 5′GTTACACCCCAATCTTCATGTCCAC3′; CD166-F: 5′TGGCAATATCACATGGTACAGG3′; CD166-R: 5′AGCCTTGGTTGTCTTGTACTC3′.

### Data Analysis

Statistical analysis was performed using Microsoft Excel 2013. The significance of the data was analyzed using a Student's t-test, and differences between two means with p<.05 (*) and p<.01 (#) were considered significant and very significant, respectively.

## Results

### BLE extract inhibited PC cell proliferation in a dose- and time-dependent manner

**Figure 1:**
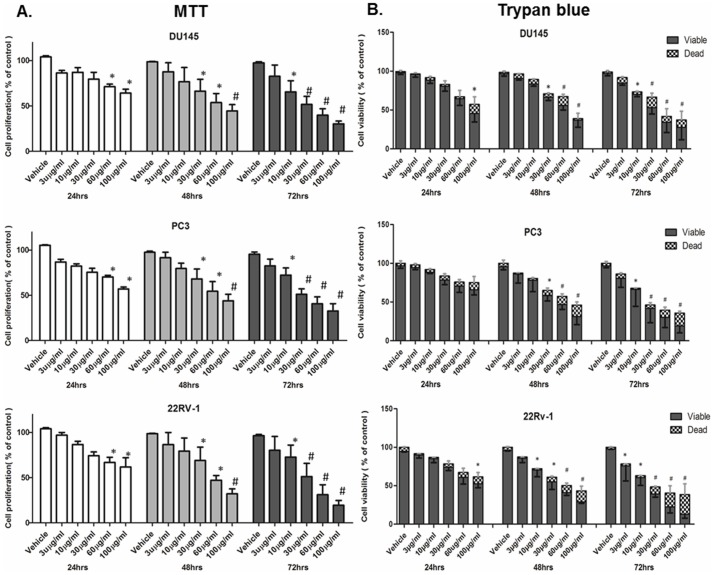
The effect of various concentrations of BLE extract on prostate cancer cell line proliferation and viability by using (A) MTT assay and (B) Trypan blue dye exclusion assay. After incubation of the three prostate cancer cell lines (DU145, PC3 and 22Rv-1) for 24, 48 and 72 h with or without treatment with BLE extract, cell proliferation was determined. Results are expressed as a percentage of the studied group compared to its control. Data represent an average of five independent experiments. The data are reported as mean ± SD (* P<0.05; # P<0.01).

Our first objective was to investigate the *in vitro* effects of the BLE extract on PC cell growth and viability. Using MTT assays (Fig. 1A), the proliferation activity of PC cell lines Du145, PC3 and 22Rv-1 was found to be inversely correlated with the extract concentration in the culture medium. After 72 h of treatment, the inhibitory effect commenced at low concentrations and significantly inhibited proliferation by 50% when PC cells were grown in media supplied with 30 µg/ml of BLE extract. However, at high extract concentration (100 µg/ml), around 75% of the anti-proliferative effect was significantly achieved. These results were compatible with confluency changes in culture (Figure S1) and verified by trypan blue exclusion assays (Fig. 1B). At high concentrations (60 and 100 µg/ml), increased cytotoxicity and cell death were observed.

### BLE extract induced cellular toxicity on DU145 cell line

**Figure 2:**
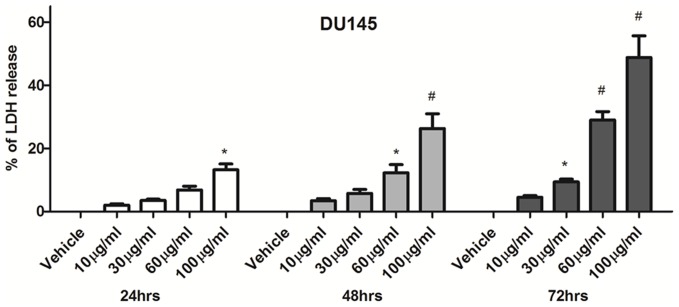
BLE extract induces cell death in DU145 prostate cancer cell lines. Cell death was determined by lactate dehydrogenase (LDH) assay treated with or without indicated concentrations of BLE extract for 24, 48 and 72 h. Cell death was calculated as the percentage of LDH release according to a previously mentioned formula. Data represent an average of three independent experiments. The data are reported as mean ± SD (* P<0.05; # P<0.01).

BLE was cytotoxic and induced significant cell death in the DU145 cell line in a dose- and time-dependent manner (Fig. 2). The cytotoxic potential of the BLE extract was determined based on the measurement of intracellular LDH enzyme, which is a stable cytosolic enzyme released into the cell culture medium upon plasma membrane damage. A 30% cytotoxic effect was significantly achieved at 48 h with 100 µg/ml and at 72 h with 60 µg/ml. However, the maximum percentage of cell death was around 50% after 72 h of treatment at a concentration of 100 µg/ml of BLE extract.

### BLE pro-oxidative activity induced DU145 cell death

**Figure 3:**
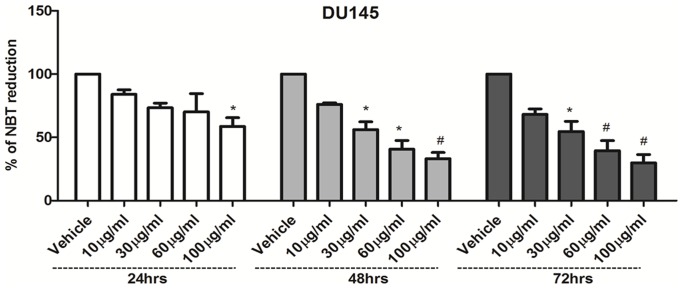
Effect of BLE extract on the production of reactive oxygen species (ROS). ROS production was measured by nitroblue tetrazolium (NBT) reduction in DU145 cell line with or without treatment with indicated concentrations of BLE extract for 24, 48 and 72 h. NBT reduction was calculated as the percentage of the control of the same group. Data represent an average of three independent experiments. The data are reported as mean ± SD (* P<0.05; # P<0.01).

Intracellular reactive oxygen species (ROS) production was measured using nitroblue tetrazolium (NBT). The intensity of the dye is inversely propotional to the presence of ROS. Results from Figure 3 indicate a dose- and time-dependent reduction of NBT. NBT reduction was initiated at 24 h with 100 µg/ml, while the maximum percentage of NBT reduction was after 72 h of treatment with 100 µg/ml of BLE extract. Interestingly, in the presence of a ROS scavenger (N-acetyl-L-cysteine, NAC), BLE failed to affect cell viability at 24 h and 48 h. Furthermore, BLE-induced cellular DNA fragmentation, indicative of apoptosis and cell death, was not seen in the presence of NAC (Fig. S2). This suggests that the BLE extract induces cell death by the induction of ROS production.

### BLE extracts induced cell cycle arrest at G0-G1 phase

In an attempt to understand how the BLE extract is able to decrease the cell count and inhibit cell proliferation, we sought to analyse the cell cycle. We examined cellular DNA content distribution by flow cytometry after 24, 48 and 72 h of treatment. Cell cycle analysis revealed that DNA content distribution is altered in BLE-treated PC3 (Table 1) and DU145 (Table 2) cells. As summarized in Table 1, BLE treatment of PC3 cells with either 10 µg/ml or 30 µg/ml caused an increase, within 24 h, in the number of cells in the G1 phase of the cell cycle, providing evidence of G1 arrest. At 48 h and 72 h post-treatment, the G1 population increased from less than 50% in the control to more than 60% in PC3 cells treated with BLE extract at 10 µg/ml or 30 µg/ml. Interestingly, this was associated with a concomitant decrease in the percentage of S phase cells and an accumulation of pre-G1 (G0) phase cells. The same profile was seen when DU145 cells were subjected to the same treatment and analysis (Table 2). These data suggest that BLE treatment results in a perturbation of the cell cycle.

**Table 1:** Summary of cell cycle analysis of PC3 cells by flow cytometry.

	PC3	Pre G0-G1	G0-G1	S	G2-M
**24 hr**	Control	1.42±0.3	46.44±1.5	21.19±1.3	30.64±2.1
	Vehicle	1.32±0.5	49.32±2.3	19.4±2.2	29.69±3.1
	10 µg/ml	1.51±0.4	57.24±2.6	16.31±3.1	24.61±3.3*
	30 µg/ml	2.22±0.2*	61.81±3.2*	11.04±1.2*	24.97±3.2*
**48 hr**	Control	1.40±0.42	47.29±4.14	18.65±1.23	32.24±2.55
	Vehicle	1.49±0.32	49.11±1.64	20.64±3.21	29.19±2.23
	10 µg/ml	2.23±1.3*	63.96±3.2*	10.85±2.92*	22.86±2.52*
	30 µg/ml	2.25±1.33*	70.49±3.24*	11.87±2.1*	15.68±2.45*
**72 hr**	Control	1.541±0.4	44.89±4.88	19.41±2.21	31.20±3.16
	Vehicle	1.69±0.53	47.78±3.33	17.87±2.14	31.18±3.21
	10 µg/ml	2.11±1.13*	59.98±3.21*	14.87±2.33*	22.73±3.12*
	30 µg/ml	2.51±1.23*	73.34±3.21*	9.44±1.22*	14.32±3.12*

Table showing the distribution of phases of the cell cycle under the effect of the BLE extract at 24, 48 and 72 h in PC3 cells. Cells that were left untreated with either vehicle or BLE (10 µg/ml and 30 µg/ml) are referred to as control. Data represent an average of three independent experiments. The data are reported as mean ± SD (Bold* P<0.05).

**Table 2:** Summary of cell cycle analysis of DU145 cells by flow cytometry.

	DU145	Pre G0-G1	G0-G1	S	G2-M
**24 hr**	Control	1.35±0.58	45.44+4.82	22.37+2.04	30.63±2.23
	Vehicle	1.34±0.34	47.95±3.84	21.63±3.03	29.08±3.96
	10 µg/ml	1.55±0.43	56.3±1.17*	18.02±2.74	24.07±2.38
	30 µg/ml	1.894±1.01*	61.36±2.53*	14.40±1.91*	22.61±2.76*
**48 hr**	Control	1.618±0.69	48.02±1.17	17.42±3.33	32.8±2.98
	Vehicle	1.75±0.92	48.27±2.36	20.64±1.91	29.34±1.63
	10 µg/ml	1.83±0.52	60.22±2.23*	14.65±2.83*	23.49±3.74*
	30 µg/ml	1.71±0.21	65.42±2.82*	14.50±1.02*	18.71±2.82*
**72 hr**	Control	1.60±0.34	44.7±4.25	21.15±2.92	32.66±2.57
	Vehicle	1.82±0.66	48.42±2.23	18.90±3.77	31.10±2.56
	10 µg/ml	2.45±0.86*	58.91±2.26*	15.43±3.23*	23.43±2.22*
	30 µg/ml	2.89±0.61*	71.27±2.74*	10.01±1.53*	16.28±1.12*

Table showing the distribution of phases of the cell cycle under the effect of the BLE extract at 24, 48 and 72 h in DU145 cells. Cells that were left untreated with either vehicle or BLE (10 µg/ml and 30 µg/ml) are referred to as control. Data represent an average of three independent experiments. The data are reported as mean ± SD (**Bold** * P<0.05).

### BLE extract greatly reduced the migration and invasion abilities of DU145 cells

**Figure 4:**
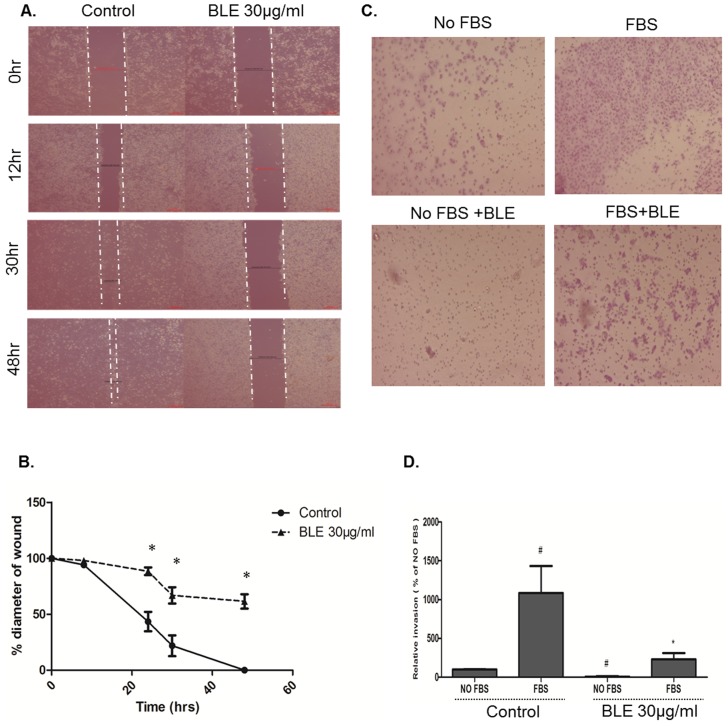
BLE extract reduces the invasive potential of human prostate cancer cells. (**A**) A scratch, in DU145 cells, was made using a yellow tip, and images were taken at T = 0 h, 12 h, 30 h and 48 h with or without treatment, and quantification of the diameter of the wound closure was assessed over time (**B**). Data represent an average of three independent experiments. The data are reported as mean ± SD (* P<0.05; # P<0.01). (**C**): representative images of trans-well invasion assay. DU145 cells were seeded onto the Matrigel™-coated membrane in the top chamber of the trans-well and were either treated or not treated with 30 µg/ml of BLE extract in the presence or absence of FBS in the lower chamber. Cells that invaded to the lower chamber after 48 h were fixed with methanol, stained with H&E, counted and represented as a percentage of invaded cells compared to the control (**D**). Data represent an average of three independent experiments. The data are reported as mean ± SD (* P<0.05; # P<0.01).

Next, we investigated the effect of the BLE extract on cell migration and invasion,, two phenotypes that are associated with the progression to metastasis. Using a wound-healing assay, in the presence of mitomycin C to inhibit cell division, BLE treatment significantly suppressed the cell migration ability of DU145 cells compared to the control, whereby the wound was completely healed after 48 h (Fig. 4A and B). These data were compatible with the invasion assay where upon treatment with 30 µg/ml of BLE extract, invasion ability, in response to FBS, was significantly reduced by more than three folds compared to the control (Fig. 4 C and D). Interestingly, BLE treatment significantly reduced the basal invasion (no FBS) of the cells when compared to the control. Moreover, and after correcting for the effect on proliferation/apoptosis, the effect of BLE on invasion remained significant between treatment and control group. This suggests that BLE extract has a high inhibitory effect on PC cell migration and invasion.

### BLE extract targeted an enriched population of PC stem/progenitor cells using 3D spheres-formation assay

**Figure 5:**
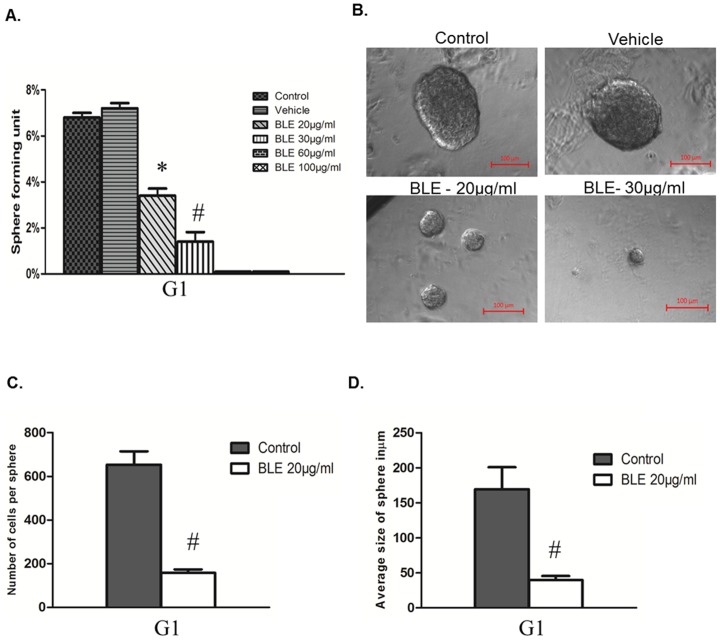
The effects of BLE extract on sphere-forming ability of DU145 cells. (**A**) Sphere-Forming Unit (SFU) is shown with or without BLE treatment (20, 30, 60 and 100 µg/ml). Generated spheres are referred to as G1 (Generation 1) spheres. SFU is calculated according to the following formula: SFU  =  (number of spheres counted ÷ number of input cells) ×100. Data represent an average of three independent experiments. The data are reported as mean ± SD (* P<0.05; # P<0.01). (**B**) Representative images of DU145 prostaspheres with or without BLE treatment. Images were visualized by Carl Zeiss microscope at 10x magnification and analyzed by Carl Zeiss Zen 2012 image software. (**C**) Quantification of the average diameter of DU145 prostaspheres with or without treatment conditions. Data represent an average of three independent experiments. The data are reported as mean ± SD (* P<0.05; # P<0.01). (**D**) Quantification of the average number of cells per DU145 prostasphere with or without treatment. Data represent an average of three independent experiments. The data are reported as mean ± SD (* P<0.05; # P<0.01).

The ability to form and propagate spheres in non-adherent culture is one of the characteristics of CSCs. To confirm that BLE treatment can inhibit prostate CSC properties, formation of prostaspheres from PC cell lines was studied in the presence or absence of BLE extract. In this assay, prostaspheres were generated by embedding 1000 single cells per well in Matrigel™ at low serum concentration (2% FBS). After culturing DU145 cells for 13 days, a low number of these cells were able to form tumor spheres in untreated conditions with a sphere-forming unit (SFU) of 6.8%. However, treatment with 20 µg/ml or 30 µg/ml of BLE extract significantly reduced the number of prostaspheres by more than 50% (Fig. 5A). In addition, no spheres were observed in wells treated with BLE extract at a concentration above 30 µg/ml. Interestingly, the average size of BLE extract-treated prostaspheres was significantly smaller compared to control (Fig 5B and D) and this could be explained by the significant reduction in the average number of cells per prostasphere as shown in figure 5C.

**Figure 6:**
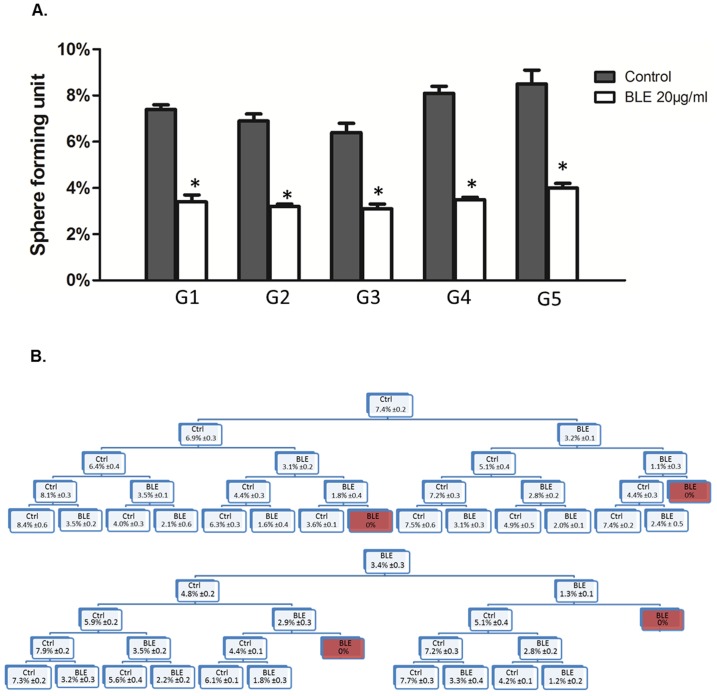
BLE treatment inhibits the self-renewal capacity of prostate cancer stem/progenitor cells. (A) SFU obtained from serially passaged prostaspheres over five generations is shown under untreated conditions (always control) and with 20 µg/ml of BLE extract-treated condition (treated once after propagation of control). (B) Prostate CSC were enriched from DU145 cell line and treated with either BLE extracts (20 µg/ml) or media (control) (G1). After each propagation, cells that were initially treated with the BLE extract (20 µg/ml) or media (control) were seeded into separate wells. Spheres were propagated for five generations in duplicates of each condition. SFU is counted and an average of two independent experiments is represented. The data are reported as mean ± SD (* P<0.05; # P<0.01).

Self-renewal activity is considered to be one of the major hallmarks of stem/progenitor cells. Thus, to ensure that our extract is able to target the self-renewing CSC pool, sphere formation activity was assessed over five generations. DU145 cells that were able to form spheres in the first generation (G1) were collected and propagated by dissociating spheres into single cells and then the same number of cells (1000 cell/well) was re-seeded. The assay was performed untill the fifth generation (G5), following an experimental design illustrated in figure 6B. Treatment with 20 µg/ml of BLE extracts was sufficient to significantly reduce the sphere formation ability to around 50% whenever treated once and consistently over five generations (Fig. 6A). Interestingly, upon successive treatment and propagation of cells that were able to resist and survive the first BLE treatment at G1, extremely demoted SFUs were produced at G2, and no prostaspheres were formed at G3. Notably, cells that were treated once at G1 were able to regain their sphere-forming ability upon further propagation of the prostaspheres in the absence of any treatment (Fig. 6B). This suggests that serial treatment with BLE extract is needed and is able to target the highly resistant CSCs, and thus extinguishing their self-renewal ability. Notably, cells treated with 30 µg/ml of BLE extract showed a significant reduction in the expression levels of Sox2, Oct4, Nanog, CD44 and CD166, all markers known to be expressed in prostate cancer stem cells, compared to control non-treated cells (Fig. S3).

## Discussion and Conclusions

The overall goal of cancer therapy is to target cancer cells only, while leaving viable normal cells unscathed. In this study, *Berberis libanotica* extract exhibited anti-neoplastic effects by reducing the viability and proliferation of three PC cell lines in a time- and dose-dependent manner, whereby the 22RV-1 cell line showed a higher sensitivity to treatment than the other two cell lines (PC3 and DU145). A concentration of 30 µg/ml of BLE extract was successful in achieving IC50, while a concentration of 60 µg/ml induced a 70% proliferation inhibition and cell death was achieved at 100 µg/ml. Several other studies that investigated the effect of alkaloids extracted from the roots of other plants such as Glaucium flavum [32], Sanguinarine [33] and Rauwolfia vomitoria [34], showed similar results in terms of their cytotoxicity and anti-proliferative effects. Previous and current work, including this study, present alkaloids as new promising agents for cancer chemotherapy.

Our data revealed a high increase in the G0-G1 peak in response to treatment with the BLE extract, suggesting an arrest at the G0-G1 phase due to the treatment. Since other studies showed that the anti-proliferative effect of alkaloid-rich plant extracts is due to G2-M arrest rather than G0-G1 arrest [35]–[37], as in the BLE extract, more studies should be done at the molecular level to better understand the altered pathways in response to BLE treatment.

The ability of cancer cells to metastasize to vital organs is the most life-threatening stage of cancer, and thus, targeting the rapidly proliferating cancer cells is critical for any chemotherapeutic drug. Unfortunately, recurrence is still observed in many patients that undergo conventional chemotherapy, such as Docetaxel [38], [39]. This is because most of these drugs are likely to target the rapidly proliferating cells in the tumor bulk, ultimately missing the quiescent or the slowly dividing CSCs. CSCs that survive chemotherapy would re-enter the cell cycle and produce highly-proliferative and rapidly-dividing progenitor cells that can re-establish the tumor. It is even probable that successive cycles of chemotherapy would strengthen a tumor by generating therapy-resistant CSCs that will give rise to a resistant progeny of rapidly proliferating cells. Therefore, it is crucial to develop a drug that affects the whole tumor, including CSCs that are found within. Recent studies highlighted novel drugs with the ability to target prostate CSCs [12]–[14]. That is why we aimed to study the effect of the BLE extract on this sub-population of stem/progenitor cells using the sphere-formation assay and the extract's ability to inhibit metastasis. Our results showed a strong inhibitory effect on the sphere-formation ability in response to BLE treatment, which is translated into a decrease in sphere size and number of cells per sphere. Interestingly, after three consecutive treatments with the same concentration of BLE extract, spheres lose their self-renewing pool (i.e. CSCs), which eventually results in their death after the 3^rd^ generation. Moreover, our results from the wound-healing and invasion assays showed a significant decrease in the ability of PC cells to migrate and invade the artificial basement membrane suggesting a role in inhibiting metastasis. These observations suggest that the therapeutic effect of the BLE extract is in its ability to target the resistive CSCs pool and ultimately prevent recurrence.

In conclusion, treatment with BLE extract at a low concentration was able to inhibit proliferation, cell migration, cell invasion and reduce viability. Moreover, the BLE extract was able to target the highly resistive CSCs population and inhibit their self-renewing ability. We believe that this one-of-a-kind approach, using the sphere-formation assay, is very useful in evaluating the potential of novel targeted therapeutics *in vitro*, which in turn, might dictate *in vivo* testing. The next step would be to evaluate the *ex vivo* efficacy of the BLE extract on primary cells extracted from human or animal PC tumors and the *in vivo* efficacy on animal models of prostate cancer.

## Supporting Information

Figure S1 **Cell Morphology and Confluency changes of prostate cancer cell lines in culture.** Representative images of (A) DU145, (B) PC3 and (C) 22Rv-1 cells were taken after 72 h in culture with or without BLE treatment. Cells were visualized by Carl Zeiss inverted image microscope at 4x magnification.
(TIF)

Figure S2 **ROS scavenger decreased the effect of BLE extract on prostate cancer cell line proliferation and DNA fragmentation.** After incubation of DU145 for 24 and 48 h, with or without 30 µg/ml of BLE extract, with one condition being in the presence of 5 mM NAC, cell proliferation (A) and DNA fragmentation (B) were determined. Results are expressed as a percentage of the studied group compared to its control. The data are reported as mean ± SD (* P<0.05).
(TIF)

Figure S3 **BLE extract reduces the expression of prostate cancer stem cell markers.** The expression levels, using qRT-PCR analysis, of SOX2, Oct4, Nanog, CD44, and CD166 were determined in DU145 cells control or treated with 30 µg/ml of BLE extract for 48 h. The values were normalized to GAPDH and expressed relative to control. The data are reported as mean ± SD (* P<0.05).
(TIF)
