# Supplementary figures and images for: Berberis libanotica Ehrenb Extract Shows Anti-Neoplastic Effects on Prostate Cancer Stem/Progenitor Cells
Source: PLoS One. 2014 Nov 7;9(11):e112453. doi: 10.1371/journal.pone.0112453 (PMC4224486; doi:10.1371/journal.pone.0112453)

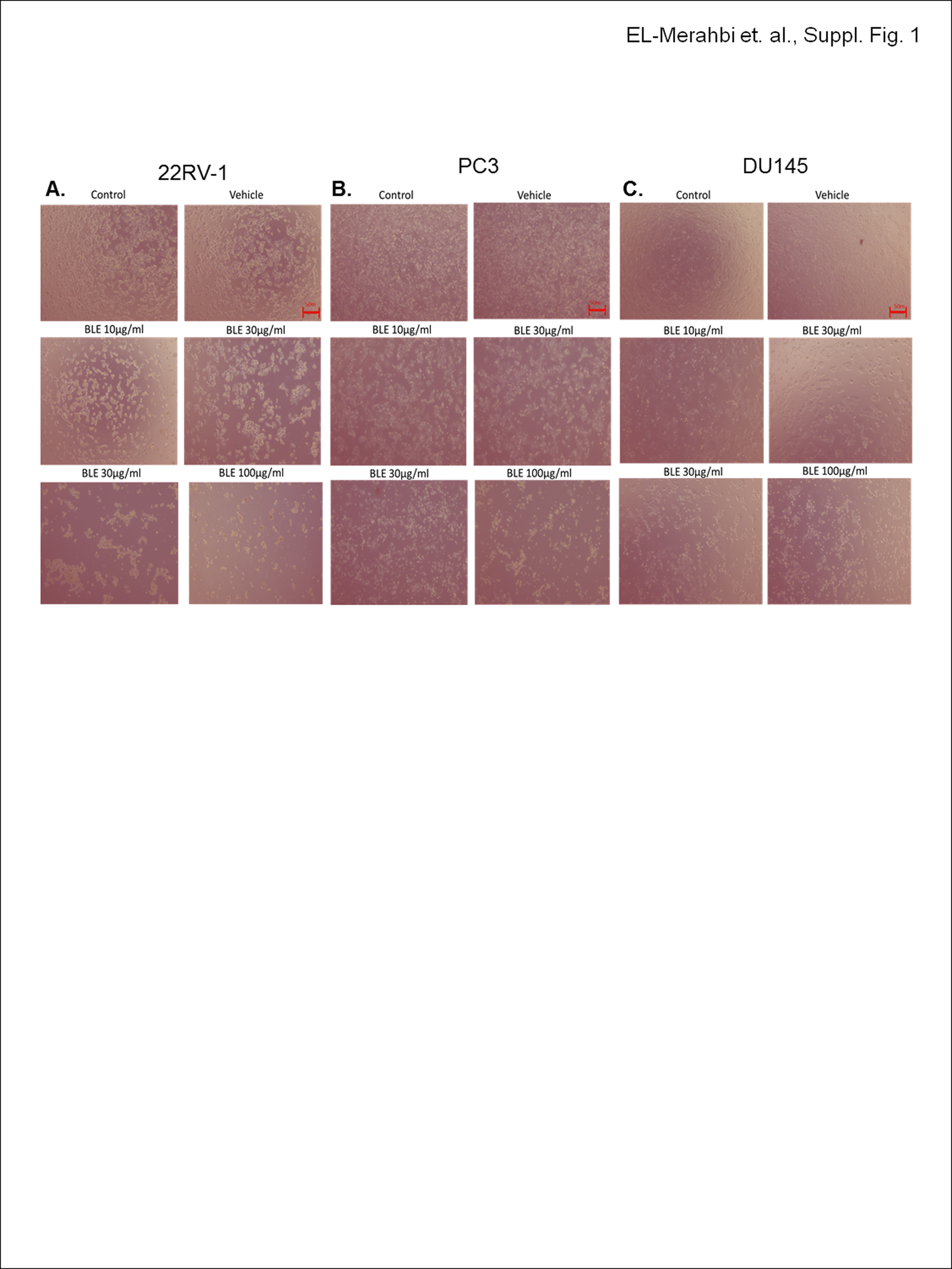

Supplement: Figure S1 — Cell Morphology and Confluency changes of prostate cancer cell lines in culture. Representative images of (A) DU145, (B) PC3 and (C) 22Rv-1 cells were taken after 72 h in culture with or without BLE treatment. Cells were visualized by Carl Zeiss inverted image microscope at 4x magnification. (TIF) [file pone.0112453.s001.tif]

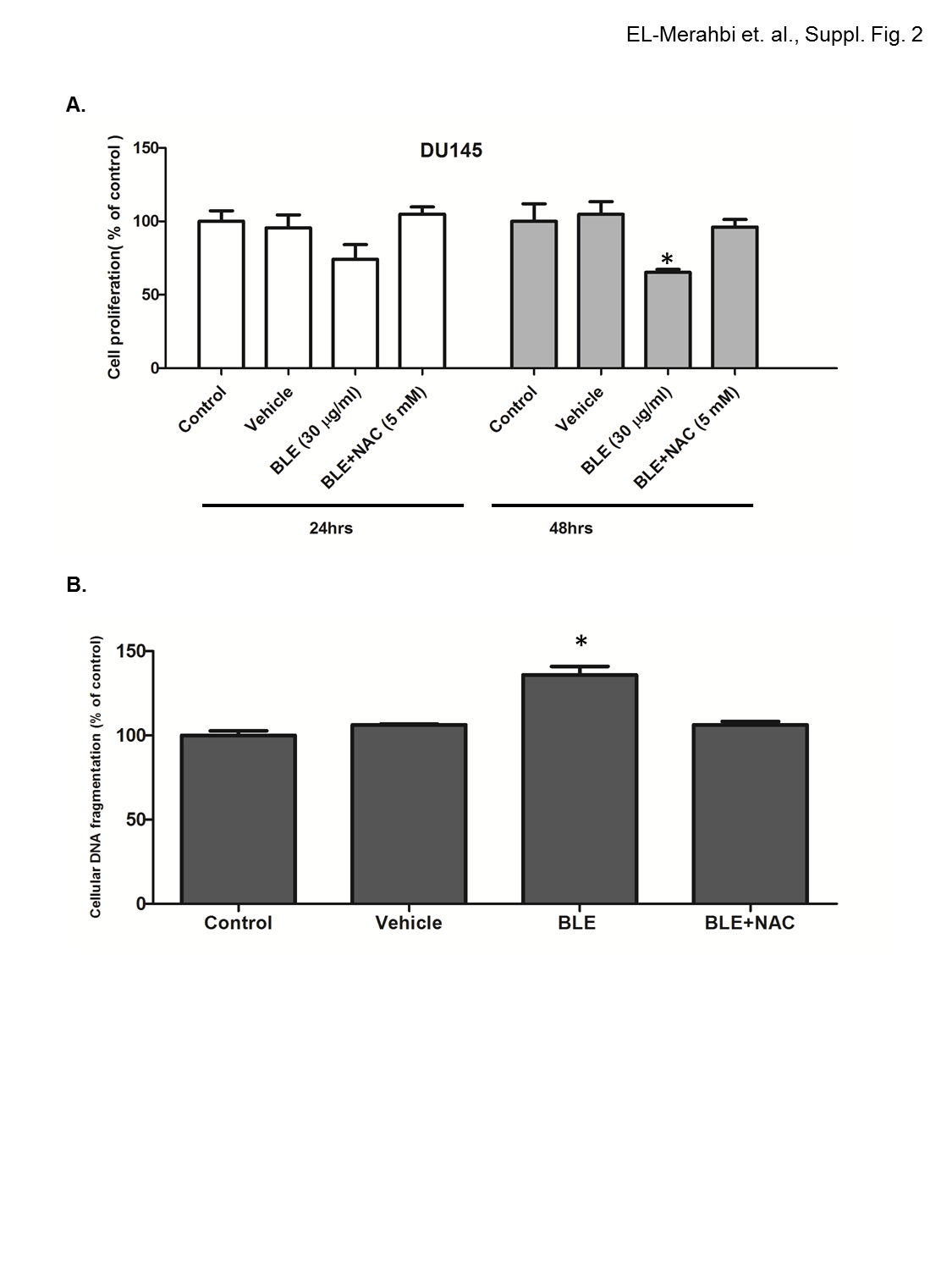

Supplement: Figure S2 — ROS scavenger decreased the effect of BLE extract on prostate cancer cell line proliferation and DNA fragmentation. After incubation of DU145 for 24 and 48 h, with or without 30 µg/ml of BLE extract, with one condition being in the presence of 5 mM NAC, cell proliferation (A) and DNA fragmentation (B) were determined. Results are expressed as a percentage of the studied group compared to its control. The data are reported as mean ± SD (* P<0.05). (TIF) [file pone.0112453.s002.tif]

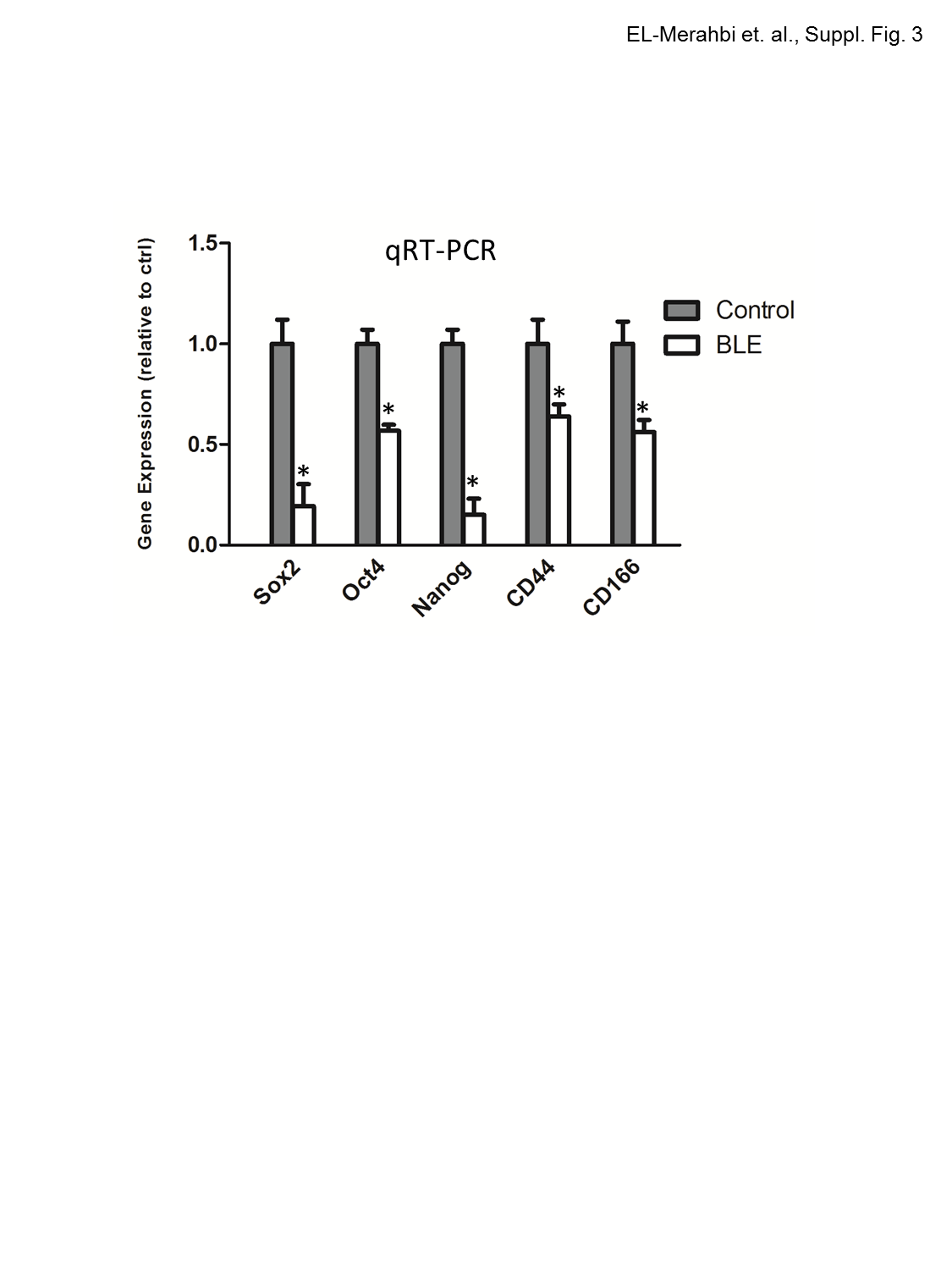

Supplement: Figure S3 — BLE extract reduces the expression of prostate cancer stem cell markers. The expression levels, using qRT-PCR analysis, of SOX2, Oct4, Nanog, CD44, and CD166 were determined in DU145 cells control or treated with 30 µg/ml of BLE extract for 48 h. The values were normalized to GAPDH and expressed relative to control. The data are reported as mean ± SD (* P<0.05). (TIF) [file pone.0112453.s003.tif]
